# Friend matters: sex differences in social language during autism diagnostic interviews

**DOI:** 10.1186/s13229-021-00483-1

**Published:** 2022-01-10

**Authors:** Meredith Cola, Lisa D. Yankowitz, Kimberly Tena, Alison Russell, Leila Bateman, Azia Knox, Samantha Plate, Laura S. Cubit, Casey J. Zampella, Juhi Pandey, Robert T. Schultz, Julia Parish-Morris

**Affiliations:** 1 Center for Autism Research, Children’s Hospital of Philadelphia, 2716 South St, Philadelphia, PA 19104 USA; 2 Department of Psychology, La Salle University, 1900 West Olney Ave, Philadelphia, PA 19141 USA; 3 Department of Psychology, University of Pennsylvania, 3720 Walnut St, Philadelphia, PA 19104 USA; 4 Department of Psychology, University of Pittsburgh, 210 S. Bouquet St, Pittsburgh, PA 15213 USA; 5 Department of Psychiatry, Perelman School of Medicine of the University of Pennsylvania, 3400 Civic Center Blvd, Philadelphia, PA 19104 USA; 6 Department of Pediatrics, Perelman School of Medicine of the University of Pennsylvania, 3400 Civic Center Blvd, Philadelphia, PA 19104 USA

**Keywords:** Autism spectrum condition, Autism spectrum disorder, Language, Social phenotype, Sex differences

## Abstract

**Background:**

Autistic individuals frequently experience social communication challenges. Girls are diagnosed with autism less often than boys even when their symptoms are equally severe, which may be due to insufficient understanding of the way autism manifests in girls. Differences in the behavioral presentation of autism, including how people talk about social topics, could contribute to these persistent problems with identification. Despite a growing body of research suggesting that autistic girls and boys present distinct symptom profiles in a variety of domains, including social attention, friendships, social motivation, and language, differences in the way that autistic boys and girls communicate verbally are not yet well understood. Closely analyzing boys’ and girls’ socially-focused language during semi-structured clinical assessments could shed light on potential sex differences in the behavioral presentation of autistic individuals that may prove useful for identifying and effectively supporting autistic girls. Here, we compare social word use in verbally fluent autistic girls and boys during the interview sections of the ADOS-2 Module 3 and measure associations with clinical phenotype.

**Methods:**

School-aged girls and boys with autism (*N* = 101, 25 females; aged 6–15) were matched on age, IQ, and parent/clinician ratings of autism symptom severity. Our primary analysis compared the number of social words produced by autistic boys and girls (normalized to account for differences in total word production). Social words are words that make reference to other people, including friends and family.

**Results:**

There was a significant main effect of sex on social word production, such that autistic girls used more social words than autistic boys. To identify the specific types of words driving this effect, additional subcategories of *friend* and *family* words were analyzed. There was a significant effect of sex on *friend* words, with girls using significantly more friend words than boys. However, there was no significant main effect of sex on *family* words, suggesting that sex differences in social word production may be driven by girls talking more about friends compared to boys, not family. To assess relationships between word use and clinical phenotype, we modeled ADOS-2 Social Affect (SA) scores as a function of social word production. In the overall sample, social word use correlated significantly with ADOS-2 SA scores, indicating that participants who used more social words were rated as less socially impaired by clinicians. However, when examined in each sex separately, this result only held for boys.

**Limitations:**

This study cannot speak to the ways in which social word use may differ for younger children, adults, or individuals who are not verbally fluent; in addition, there were more autistic boys than girls in our sample, making it difficult to detect small effects.

**Conclusions:**

Autistic girls used significantly more social words than boys during a diagnostic assessment—despite being matched on age, IQ, and both parent- and clinician-rated autism symptom severity. Sex differences in linguistic markers of social phenotype in autism are especially important in light of the late or missed diagnoses that disproportionately affect autistic girls. Specifically, heightened talk about social topics could complicate autism referral and diagnosis when non-clinician observers expect a male-typical pattern of reduced social focus, which autistic girls may not always exhibit.

**Supplementary Information:**

The online version contains supplementary material available at 10.1186/s13229-021-00483-1.

In this paper, our terminology is drawn from World Health Organization definitions, such that the word “sex” refers to genetic makeup, and “gender” refers to a socio-cultural construct [1]; we use the words “girl” and “boy” to refer to sex as reported by parents or caregivers. We acknowledge that the concepts of sex and gender are not binary and recognize that many autistic individuals identify as transgender, non-binary, or gender diverse [2, 3]. In the current study, participant sex was characterized using parent-reported assigned sex at birth. We recognize this approach does not account for individuals who identify as transgender, non-binary, or gender diverse and acknowledge the limitations of this methodology. In line with preferences expressed by some self-advocates, parents, and caregivers within the autistic community [4–6], this paper uses identity-first language (i.e., autistic girls and boys). Further, based on journal usage guidelines informed by stakeholders in the autism community, we refer to autism spectrum disorder (ASD) as autism throughout this manuscript [7].

## Introduction

Autism is a complex, heterogeneous neurodevelopmental condition that affects 1 in 54 children [8], and is characterized by social communication difficulties and restricted and repetitive patterns of behaviors and interests [9]. The majority of autistic individuals acquire spoken language [10, 11], but nonetheless face a wide range of challenges, including atypical conversational skills [12–14]. Recent research suggests that autistic girls may converse differently than autistic boys, resulting in better first impressions during “get-to-know-you” conversations [15]. These potential sex differences in verbal social communication—combined with male-referenced diagnostic criteria and unequal societal expectations for boys’ and girls’ social interaction skills across development—may factor into the late or missed diagnoses that are more common for autistic girls and women than boys and men [8, 16–18]. For autistic girls and women, late or inaccurate diagnoses mean missing out on evidence-based interventions, reduced access to social supports, and increased likelihood of experiencing social rejection, sexual abuse, and poor mental health outcomes [19–21]. Despite a growing body of research on sex-differentiated profiles of social attention [22, 23], gesture [24], imaginative play [25], friendships [26, 27], social motivation [28], social reciprocity [29], and language [30–35] in autism, differences in the way that autistic boys and girls communicate verbally are not yet well understood. Characterizing similarities and differences in the language produced by verbally fluent autistic boys and girls—particularly with regards to social topics in naturalistic contexts—could shed light on sex-specific differences in the behavioral presentation of autism. A specific focus on characterizing verbal communication patterns in autistic girls and boys during diagnostic assessments with clinicians could improve diagnostic accuracy and ultimately inform the development of personalized supports that are tailored to the needs of autistic girls and women.

### Language in autism

Language is a complex social phenomenon that mediates how individuals approach and operate within their social worlds [36]. More than just a system of communication with receptive and expressive components, language can be understood as a form of identity construction, social action, and a mode of experience [37]. For verbal autistic individuals, as for others, language in the context of social communication forms a critical pathway to friendships, romantic relationships, jobs, and overall quality of life [12].

Social communication challenges are core to the autism diagnosis, despite substantial within-diagnosis heterogeneity [9]. As an umbrella term, social communication includes an array of verbal and nonverbal behaviors including the use of appropriate language in a social context (i.e., pragmatic language [38]). Pragmatic language impairments have been noted in autism since the earliest descriptions of the condition [39] and socially-focused language produced by verbally fluent autistic individuals provides a key window into the workings of the social mind [40]. Words, in particular, may be especially informative. Words are used to convey experiences and ideas to other people, and word choice and relative frequency can highlight what a speaker finds important enough to describe [41–43]. Within autism, word choice has been argued to be a measure of social attention or cognition [40, 44], and research shows that autistic individuals talk less about social topics than neurotypical (NT) peers during experimental tasks [45–47]. Notably, prior research on social language or word choice in autism has not included adequate numbers of autistic girls and women to examine potential sex differences in this domain (i.e., studies either did not report participant sex or samples were approximately 85% male).

### Friendship in autism

Humans use language to achieve a complex set of social goals, including meeting diverse situational demands and conforming to societal expectations – which often differ by sex [48]. Friendship is one area where the differential experiences of autistic girls and boys are just beginning to be understood, and where word-based differences in the way individuals talk about friendships could prove informative. Challenges associated with establishing or sustaining peer relationships are frequently observed in autistic individuals, but research suggests many autistic people are nonetheless interested in making and maintaining friendships [26, 28, 49–53]. Studies of sex differences in friendship and peer conflict show that autistic girls and boys have quantitatively distinct experiences and that these differences largely mirror reported sex differences in neurotypical development. Thus, the social “worlds” of girls and boys may be qualitatively different whether or not they have an autism diagnosis [54, 55]. Whereas autistic girls rate their friendships as close, secure, and based around emotional sharing and spending time together, autistic boys report that their friendships are more casual and centered around shared activities or interests, such as video games [27, 51, 53, 56]. In adolescence, autistic girls report greater friendship quality than autistic boys, with quality levels approaching those found in NT girls [27, 28]. Notably, autistic individuals also experience friendship challenges that differ by sex. Research that assessed social challenges using the Revised Peer Experiences Questionnaire [57] found that autistic girls report experiencing more relational conflict with peers (e.g., “I was left out of a group activity”) while boys report more overt difficulties (e.g., “Someone threatened to hurt me or beat me up”) [27]. Autistic girls generally view friendship as desired [27], important and rewarding [58], but difficult to maintain [27, 50]. This suggests that culturally-gendered expectations about the importance of social relationships may play a critical role in autistic individuals’ social views and experiences (i.e., social acceptance may be judged as very important for girls in a society that rewards their relational competence and less important for boys in a society that values their independence) [59].

Previous work has identified a potential disconnect for autistic girls in social domains, including friendship, such that autistic girls and women may appear more socially competent than they actually are [21, 60]. For example, teachers report substantially fewer concerns about social skills in school-aged autistic girls compared to boys, in part because girls “blend in” or “camouflage” with peers at the surface level of observed behavior [61] despite internal struggles that ultimately increase their risk of developing anxiety or depression [50, 62]. Autistic girls are also more likely to be accepted by non-autistic girls as fringe members of female social groups until adolescence when female friendships evolve and begin to require considerably more nuanced social skills [63, 64]. Interestingly, mixed-methods research examining adolescents’ motivation for using camouflaging techniques to mask their autistic behaviors has revealed that “making or keeping friends” was the most common theme reported for both autistic girls and boys [50]. Notably, some researchers have criticized previous studies of camouflaging due to inconsistent operational definitions and imprecise measures [65]. To this end, measuring sex differences in language during conversation could provide an objective and fine-grained measure of what it might look like for autistic girls and boys to “blend in” linguistically or not.

### Population-level sex differences in talking about friendship

Within neurotypical development, it has been argued that on average, girls demonstrate better social skills and improved socio-cognitive functioning compared to boys [66, 67]. The extant literature suggests that throughout childhood and adolescence, girls are able to generate and maintain friendships and intimate relationships more readily than boys [68, 69]. The heightened social abilities of girls and women are reflected in both their written and spoken language, which contain more words related to psychological and social processes, than the language of boys and men, who refer more to object properties and impersonal topics [48, 70]. Analysis of third-party ratings has shown that “female-typical” language tends to be rated as more socially positive and accommodating than “male-typical” language in both adults [71–73], and children [74], reflecting higher levels of social intelligence. Interestingly, the effect sizes of sex differences in social language tend to be larger in less structured, conversational tasks [70] compared to monologic elicitations like narratives, highlighting the potential of investigating this phenomenon in the context of dyadic interactions. Although there is emerging research to suggest that differences in social motivation (or social focus) may be detectable in natural language samples of unstructured conversations in autism [34], this topic has only been minimally explored in a semi-structured interview context. Understanding similarities and differences in the language produced by verbally fluent autistic boys and girls—particularly regarding social topics—could shed light on sex-specific differences in the behavioral presentation of autism that may prove useful for identifying and effectively supporting autistic girls. Three facts motivate this research: (1) social communication is a core diagnostic component of autism [9]; (2) autistic girls and boys are socialized differently from birth [27, 51]; and (3) population-level sex differences exist in a variety of social-linguistic domains that may or may not be preserved in autism [75, 76]. In this study, we ask whether autistic girls and boys speak differently about social topics during a research-reliable administration of the ADOS-2.

#### The Autism Diagnostic Observation Schedule—2nd edition (ADOS-2)

Language samples for the current study were drawn from research-reliable administrations of the ADOS-2 Module 3 [77]. The ADOS-2 is a semi-structured, standardized assessment of communication, social interaction, play/imaginative use of materials, and restricted and repetitive behaviors that is designed to assess autism symptoms in verbally fluent individuals aged 4–15 years. Although both verbal and nonverbal behaviors are assessed during the ADOS-2 Module 3, it is largely a language-mediated measure. Notably, the norming sample used in the development of the ADOS-2 was predominantly male [78], leaving open the possibility that clinically meaningful sex differences in the behavioral presentation of autistic individuals went undetected or were judged unimportant for inclusion in the final algorithm. As such, it is critical to understand potential sex differences in the social language of girls and boys on this measure. The school-age period is especially important for understanding sex differences among autistic individuals without co-occurring intellectual disability (ID), as many of these individuals, particularly girls, are first diagnosed during this time [79, 80]. Due to the field's heavy reliance on the ADOS-2 for both diagnostic and research purposes, understanding sex differences in children’s behavioral presentation during this assessment could alleviate diagnostic disparities and facilitate opportunities for support and intervention.

To date, a handful of studies have used computational or word frequency-based approaches to examine language produced during the ADOS-2 in autistic school-age youth (in addition to research using qualitative coding [81]). These studies focused primarily on lexico-semantic aspects of language including disfluencies [33], sentiment and linguistic abstraction [82], nouns versus cognitive process words [30], latent semantic similarity [83], number of word roots and content maze repetition [84], and acoustic-prosodic features [85–87]. However, children’s use of social words more broadly during the interview sections of the ADOS-2 has not been explored, and critically, only two prior studies included large enough samples of autistic girls or women to examine potential sex differences [30, 33].

### Current study

In this study, we investigate sex differences in the behavioral presentation of autistic individuals by examining social word production in age and IQ-matched girls and boys during the interview sections of a commonly used diagnostic assessment, the ADOS-2 [77]. Social words were defined as words that make reference to other people (e.g., “classmates,” “everyone,” or “them”). We specifically examined sex differences, because although a literature on social language in autism exists, it is currently unclear whether social word use differs for *all* autistic individuals, since many prior studies of social word use in autism included few—if any—girls and women. Our primary hypothesis was that autistic girls would use more social words than autistic boys (marking potentially increased social motivation, greater attentional focus on social groups, and/or camouflaging, but not necessarily greater social skill). This hypothesis was informed by previous research demonstrating sex differences in social motivation in autism (girls > boys) [28] and emerging research suggesting that autistic girls produce more socially-focused language than boys during narratives and unstructured interactions [30, 34]. However, this question has never been explored in the context of the interview sections of the ADOS-2. To understand potential differences in social word use at a fine-grained level, we analyzed two subcategories of social words—*friend* and *family* words—to assess whether either type of word drove observed differences in social talk. *Friend* words are words that make reference to friends or peers (e.g., “buddies” or “best friend”). *Family* words are words that make reference to various family members (e.g., “mom” or “brother”) [43]. We hypothesized that autistic girls would demonstrate greater relative use of *friend* words compared to autistic boys, because prior research suggests that autistic girls value friendship more than autistic boys [26, 28, 88]. We did not hypothesize sex differences in the use of *family* words, as no studies to date have shown sex differences in the familial relationships of autistic boys and girls. Finally, we hypothesized that social word use would correlate with social phenotype, such that greater social word use would be associated with fewer autism symptoms as rated by a clinician.

## Methods

### Participants

One hundred and one autistic participants (*N* = 101, 25 females) and thirty-four NT participants (*N* = 34, 14 females) aged 6–15 years old were selected from a pool of verbally fluent individuals who were seen at a large academic medical research center (Children’s Hospital of Philadelphia Center for Autism Research). Verbal fluency was defined by an individual’s ability to demonstrate regular use of complex sentences, expressive language skills at or above a typical four-year-old level, produce a range of sentence types and grammatical forms, provide information beyond immediate context, and use logical connections such as “but” and “because” [77]. Participant sex was characterized using parent-reported assigned sex at birth. Children participated in a larger series of studies that included autism diagnostic assessments, IQ testing, and behavioral tasks. To match groups, participants with complete data (age, sex, race, ADOS-2 Module 3 recordings, and IQ testing) were first selected from the larger pool. Participants from the larger pool were excluded from the present analyses if they had a FSIQ or VIQ ≤ 70. Autistic and NT participants were matched group-wise on average age and IQ. Autistic girls and boys were matched on average age, IQ, and autism symptom severity at the group level, as measured by Autism Diagnostic Observation Schedule—2nd Edition (ADOS-2 [77]) scores and Social Communication Questionnaire (SCQ) “Lifetime” version [89] scores. After group-level matching on the above variables, boys and girls did not differ on Vineland Adaptive Behavior Scales, 2nd edition (VABS) communication or socialization subdomain scores [90]. Participant characteristics and matching statistics are provided in Table 1.

**Table 1:** Demographic and clinical characteristics of participants (means, standard deviations, and ranges)

	Females (*N* = 25)	Males (*N* = 76)	Effects
Race	Black or African American: 1 White/Caucasian: 21 Asian or Pacific Islander: 1 Multiracial: 2	Black or African American: 5 White/Caucasian: 63 Asian or Pacific Islander: 2 Multiracial: 6	χ^2^ = 1.99, *p* = .57
Maternal education	High school or less: 0.04% (*n* = 1) Bachelor’s or less: 36% (*n* = 9) Graduate degree: 40% (*n* = 10) Not reported: 20% (*n* = 5)	High school or less: 5.3% (*n* = 4) Bachelor’s or less: 60.5% (*n* = 46) Graduate degree: 31.6% (*n* = 24) Not reported: 2.6% (*n* = 2)	χ^2^ = 2.14, *p* = .34

			Effect of sex
Age (years)	10.66 (1.59) 8.6–14.1	10.15 (2.15) 6.1–15.1	*p* = .29 *d* = -.25
Full-Scale IQ	106.24 (11.88) 78–131	103.71 (14.83) 73–148	*p* = .44 *d* = − .18
Verbal IQ	106.64 (12.86) 79–134	105.00 (14.64) 71–150	*p* = .62 *d* = -.12
Non-verbal IQ	106.24 (13.76) 73–133	103.70 (14.87) 72–143	*p* = .45 *d* = − .17
ADOS-2 Total	10.92 (5.04) 3–23	11.89 (4.64) 4–24	*p* = .39 *d* = .20
ADOS-2 SA Total	8.28 (4.19) 3–17	9.21 (4.06) 3–19	*p* = .33 *d* = .23
ADOS-2 RRB Total	2.64 (1.87) 0–7	2.66 (1.65) 0–7	*p* = .96 *d* = .01
SCQ Total	19.96 (5.95) 8–31	19.29 (7.23) 5–38	*p* = .68 *d* = -.26
SRS-2 Total	78.04 (9.92) 57–91	71.01 (11.29) 46–90	*p* = .009* *d* = − .64
SRS-2 Social Awareness	74.48 (9.54) 58–90	68.36 (10.78) 45–90	*p* = .02* *d* = − .58
SRS-2 Social Cognition	74.04 (12.61) 49–90	67.99 (10.61) 48–90	*p* = .03* *d* = − .54
SRS-2 Social Communication	77.43 (10.57) 52–90	69.84 (11.90) 45–90	*p* = .008* *d* = − .65
SRS-2 Social Motivation	71.65 (11.64) 51–90	65.44 (11.92) 40–103	*p* = .03* *d* = − .52
SRS-2 Restricted Interests and Repetitive Behaviors	77.13 (12.16) 50–98	71.03 (11.98) 46–90	*p* = .03* *d* = − .51
SRS-2 Social Communication and Interaction	77.30 (9.78) 56–90	70.31 (11.12) 46–90	*p* = .009* *d* = − .65
VABS Communication Standard Score	87.40 (12.21) 65–108	86.92 (13.52) 62–125	*p* = .88 *d* = − .04
VABS Socialization Standard Score	73.60 (11.92) 58–112	77.22 (14.92) 36–119	*p* = .25 *d* = .27

ADOS-2 SA = Social Affect Domain Score; RRB = Repetitive Behaviors/Restricted Interests Domain Score

Chi-squared tests with Yates’ continuity correction were used to test for diagnostic group differences in sex ratio and maternal educational attainment. *p* values and Cohen’s *d* values for main effect of sex in the autism  group are shown.

Participants were recruited using a variety of methods, including public advertising, word-of-mouth, and re-recruiting from previous studies. Participants were excluded if they had a known genetic syndrome, history of concussion or brain injury that impacted current functioning, history of medication use that caused permanent changes in motor behavior (e.g., amphetamines), gestational age below thirty-four weeks, or if English was not their primary language. Parents of participants provided written informed consent to participate in this study, which was overseen by the Children’s Hospital of Philadelphia Institutional Review Board.

### Measures

All participants completed the ADOS-2 Module 3 [77], a clinician-administered assessment of the presence and severity of autism symptoms. Participants received Module 3, which requires fluent verbal skills, depending on their chronological age and the examiner’s clinical judgment. Overall scores were calculated for the domains of Social Affect and Restricted and Repetitive Behaviors [91]. Parents and caregivers completed the Social Communication Questionnaire (SCQ [89]) to assess the presence of autism symptoms. Autism diagnoses were made by expert PhD-level clinicians using the clinical best estimate (CBE) approach [92]. The CBE method prioritizes DSM-5 criteria informed by family/medical history and an evaluation by an autism specialist. The Center for Autism Research does not rely solely on ADOS-2 or SCQ cutoff scores when diagnosing autism, nor do subthreshold scores lead to automatic exclusion. This is because many disorders can result in elevated scores on these metrics (e.g., ADHD [93]), and the behavior snapshot afforded by the ADOS-2 may not capture the full scope or severity of an individual’s symptoms.

All participants received a cognitive assessment. Clinicians administered either the Differential Ability Scales-2nd Edition (DAS-II [94]), the Wechsler Abbreviated Scale of Intelligence-2nd Edition (WASI-II [95]), the Stanford-Binet Intelligence Scales-5th Edition (SB5 [96]), or the Wechsler Intelligence Scale for Children-5^th^ Edition (WISC-V [97]), according to the protocol of the larger study from which the current sample was drawn. To allow for comparison across these assessments, scores were standardized and reduced to an overall cognitive estimate (Full-Scale IQ), as well as Verbal IQ and Nonverbal IQ subscores by an expert licensed neuropsychologist (J. Pandey).

Additionally, parents completed the Vineland Adaptive Behavior Scales, 2nd edition (VABS [90]) parent-caregiver form to assess adaptive behavior in the domains of communication and socialization. The Vineland is a sex-normed and age-normed measure that assesses adaptive behavior skills in individuals from birth to age 90 and divides adaptive behavior into three broad domains. Standard scores are generated for each domain.

### Language sample

Linguistic data were drawn from the interview sections of research-reliable administrations of the ADOS-2 Module 3, recorded at the Center for Autism Research at the Children’s Hospital of Philadelphia. For the purpose of these analyses, linguistic data from the following ADOS-2 sections were included: emotions, social difficulties and annoyance, friendships, relationships and marriage, and loneliness. These conversations provide a rich, dyadic semi-structured language sample that includes discussion of diverse social topics. Breaks were not included in analyses. Conversations were audio/video recorded using standard free-standing video cameras. Total length of the conversation did not differ by participant sex (estimate: − 0.05, SE: 1.25, *p* = 0.97; overall mean = 21.7 min, overall SD = 6.14 min).

### Data processing

Audio recordings of each conversation were orthographically transcribed by reliable annotators who were unaware of the participants’ diagnostic status and study hypotheses. Annotators were undergraduate student research assistants, trained on a modified Quick Transcription protocol for XTrans software [98, 99]; all were trained on segmenting and transcription, with a minimum 92% word-level reliability criteria that must be met consistently before beginning to transcribe [100]. Both junior and senior annotators worked on the transcription process. Junior annotators were allowed to segment or transcribe, but only senior annotators with at least six months of XTrans transcription experience were allowed to check and approve final transcripts. As part of a standard transcription pipeline, multiple annotators (student workers) processed each transcript: the first annotator segmented speech into pause groups (generally 6–8 s long) and labeled each segment as coming from either the participant or the clinician; the second and third annotators independently transcribed words and sounds produced by speakers. After this, in-house R and python scripts were run to generate a differences file, which identified any segments with transcription discrepancies. All files were transcribed by two independent annotators, with pre-adjudication word-level agreement averaging 92.97%. Finally, a senior annotator reviewed the differences file and adjudicated any discrepancies to produce the final file. After the process of adjudication was completed, the final files were converted to basic text format, imported into R, and processed for analysis using the qdap package [101]. Text files were fed into LIWC software [102], which calculated the overall number of words produced, as well as the number of friend, family, and social category words produced by participants (see *Dependent variables,* below).

#### Statistical approach

Data were analyzed using generalized linear regression models (GLM) in R (‘lme4’ package; R Core Team and contributors worldwide) with age and IQ (mean centered) as covariates. Estimated effects, standard errors (SE), *z*-values, and *p*-values are provided. Variables were coded as female = 0, male = 1. Models used in the present analyses were tested progressively and selected using fit statistic parameters (AIC). Dependent variables were positive, interval, and non-normally distributed (Shapiro–Wilk test *p*s < 0.001), so these data were modeled using a Poisson distribution with a log link. Significance values for planned pairwise tests of GLM estimated marginal means were corrected for multiple comparisons using the Tukey method. Effect sizes for GLM are reported as unstandardized effects (estimates [103]), while Cohen’s *d* is reported for group mean differences on clinical and demographic variables (Table 1). Following Cohen [104], *d* = 0.2 is considered a “small” effect, *d* = 0.5 a “medium” effect, and *d* = 0.8 a “large” effect. GLMER was used to assess relationships between social words production and clinical phenotype (ADOS-2 Total, Social Affect, and Restricted Repetitive Behaviors domain total scores).

#### Dependent variables

Preliminary analyses controlling for age and IQ (mean centered) revealed that girls produced, on average, 200 more words than boys during the interview  sections of the ADOS-2 (see Table 2). Thus, subsequent analyses were conducted on the number of social category words (e.g., “person”, “everyone”), friend category words (e.g., “buddies”, “best friend”), and family category words (e.g., “mom”, “brother”), as calculated by LIWC, normalized per 1000 words to account for individuals’ varying word production. We decided to normalize word use per 1000 words based on the average range of words produced by participants in our study and to illustrate relative frequency without reporting percentages that could be misinterpreted when participants produced fewer overall words. We further avoided the use of proportions because they tend to violate the underlying assumptions of common statistical tests, can be misleading when the number of words produced varies widely (as in this study and most studies of productive language in autism), and do not generally adhere to the way words are counted (usually full words are counted as words, and thus are better represented as count data than as decimals; counts per 1000 words were therefore rounded to the nearest whole number). Clinical phenotype was measured using ADOS-2 Total, Social Affect, and Restricted Repetitive Behaviors domain total scores.

**Table 2:** Characteristics of participant speech by sex (means, standard deviations, and ranges)

	Females	Males	Effects
*Participant speech behavior*			
Part A			
Social word frequency per 1000 words	127.04 (24.8) 59–161	110.59 (24.3) 59–171	*p* < .001** est: − .13
Friend word frequency per 1000 words	7.72 (4.92) 2–23	6.13 (3.81) 0–15	*p* = .01* est: − .22
Family word frequency per 1000 words	8.88 (5.84) 0–26	8.32 (5.61) 0–28	*p* = .18 est: − .11
Part B			
Total length of conversation (min)	21.86 (4.24) 14.3–38.7	21.64 (6.67) 10.8–52.3	*p* = .97 est: − .05
Total time speaking (min)	7.66 (3.29) 2.9–15.4	6.96 (3.59) 0.8–19.3	*p* = .15 est: − .87
Word count	1218.72 (545.97) 318–2420	1024.68 (544.47) 132–3091	*p* = .11 est: − 150.9
Characters per word	3.83 (0.11) 3.6–4.1	3.77 (0.15) 3.4–4.1	*p* = .18 est: < .001
Type-token ratio	0.40 (0.08) .30–.63	0.37 (0.07) .24–.60	*p* = .05 est: < .001

Effect sizes for GLM are reported as unstandardized effects (estimates [94]). The final GLM model [glm(variable ~ age.z + IQ.z + sex, data = lang.par, family = ‘poisson’)] accounts for age (centered), IQ (centered), and examines sex as primary predictor variable. Part A includes the primary variables of interest. Part B includes additional variables used to characterize the language sample. Effect of sex is significant *p* < .01

#### Preliminary analyses

To ensure that participant groups did not differ on basic metrics of structural language, we compared girls and boys on three features beyond of our dependent variables of interest: characters per word, type-token ratio (a measure of lexical diversity), and length of time spent speaking (Table 2, Part B). Results showed that boys and girls were broadly comparable on these language metrics, in addition to having comparable verbal IQ scores, VABS communication and socialization scores, and autism symptom severity as rated by parents and clinicians.

## Results

### Social words

**Fig. 1:**
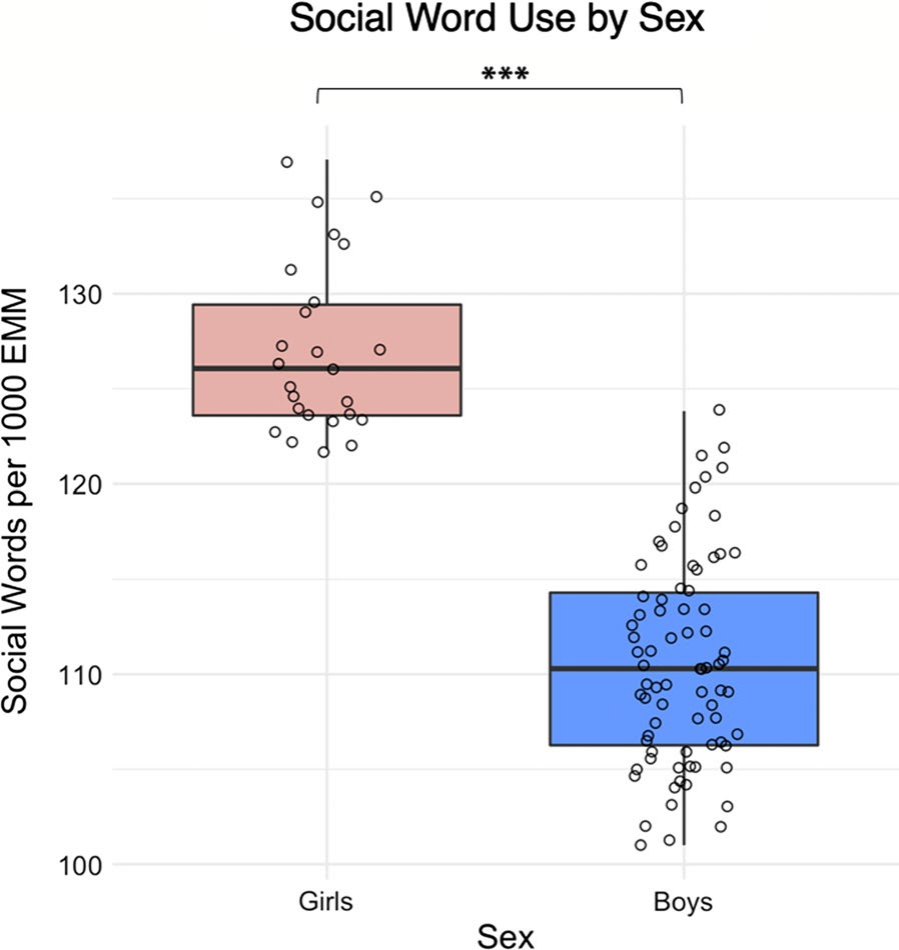
Estimated marginal mean social word use per 1000 words by sex after accounting for age (centered) and IQ (centered)

A generalized linear regression model predicting participant social word production revealed a significant main effect of sex (estimate: − 0.13, SE: 0.02, *z* = − 6.07, *p* < 0.001). The model controlled for age (centered) and IQ (centered). Tukey-corrected pairwise comparisons of estimated marginal means revealed that the effect was driven by girls producing more social words than boys (Fig. 1; Table 2). There was also a conditional effect of age (after accounting for sex) on social word production (estimate: 0.05, SE: 0.01, *z* = 4.89, *p* < 0.001), with older participants producing more social words than younger participants. The effect of IQ on social word production was not significant (estimate: − 0.13, SE: 0.02, *z* = − 6.07, *p* = 0.79). To further examine the kinds of social words being used, subcategories of *friend* and *family* words were analyzed.

#### Friend and family words

**Fig. 2:**
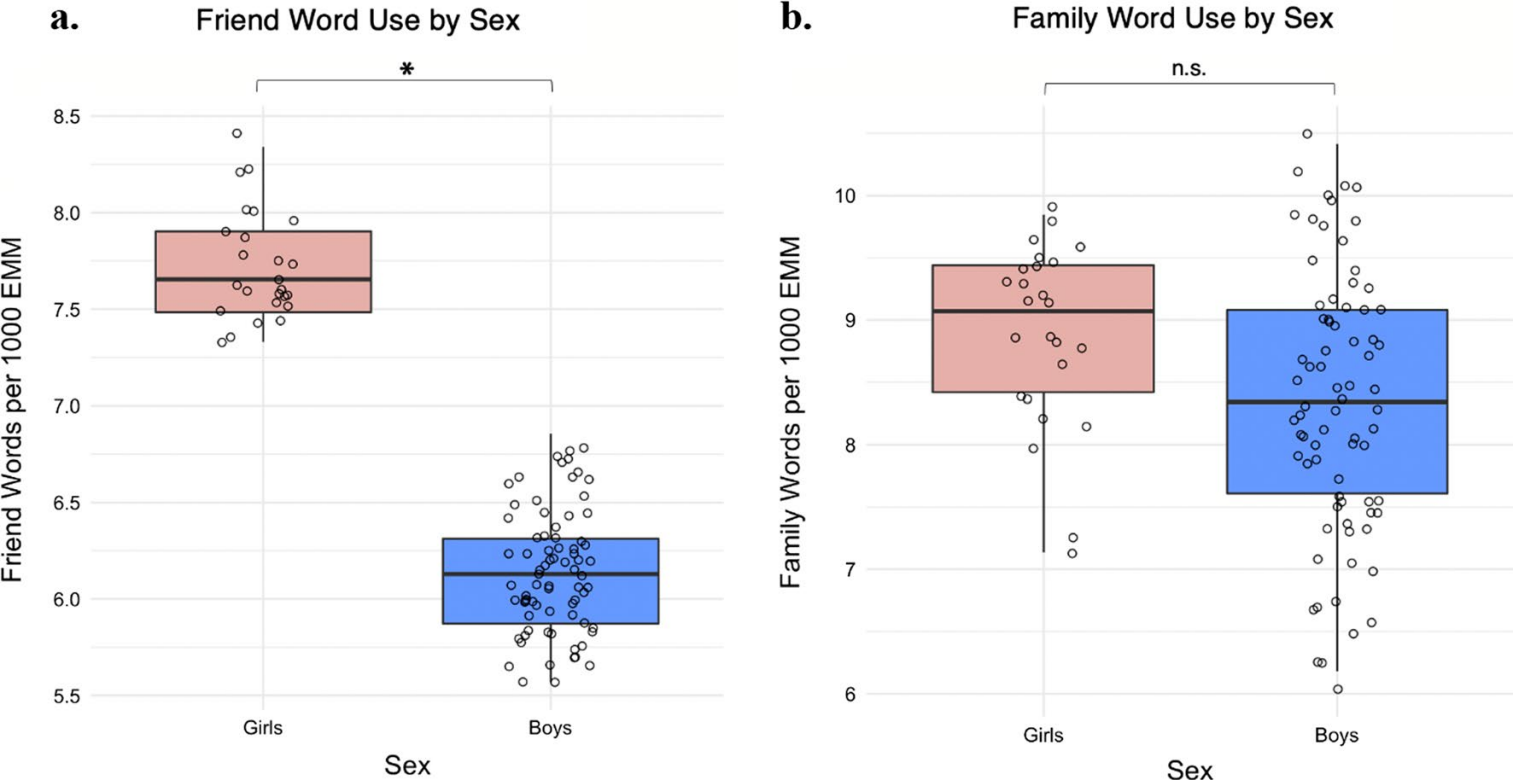
Estimated marginal means friend and family word use per 1000 words by sex after accounting for age (centered) and IQ (centered)

A GLM including age (centered), IQ (centered), and sex revealed a significant effect of sex on *friend* words (estimate: − 0.22, SE: 0.09, *z* = − 2.55, *p* = 0.01), with girls using more friend words than boys (see Fig. 2a; Table 2). A separate GLM predicting *family* words after controlling for age (centered) and IQ (centered) revealed no significant effect of sex on family words (Fig. 2b; estimate: − 0.11, SE: 0.08, *z* = − 1.33, *p* = 0.18). Taken together, these results suggest that sex differences in social word production were driven in part by girls talking more about friends than boys, but not family (see Table 2).

#### Predicting clinician-rated phenotype

To determine whether social word production was associated with clinician-rated phenotype in autism, we modeled ADOS-2 social affect (SA) total scores as a function of social word production. After accounting for age (centered) and IQ (centered), social word production significantly predicted ADOS-2 SA total scores in the overall sample (estimate: − 0.005, SE: 0.001, *z* = − 3.58, *p* < 0.001), indicating that participants who used fewer social words were rated as more socially impaired by expert clinicians. In an exploratory analysis performed in each sex separately, we found that social word production predicted ADOS-2 SA scores for boys (estimate: − 0.005, SE: 0.002, *z* = − 3.13, *p* = 0.002), but not for girls (est: − 0.003, SE: 0.003, *z* = − 1.02, *p* = 0.31). As illustrated by Fig. 1, this may be due to reduced power in smaller sex-based subsamples, or to restricted range in girls (100% of girls produced more social words than 97.4% of boys, with a smaller range). Alternatively, as suggested by recent research [34], social language output may be relatively abundant but atypical in girls, and thus the number of social words produced may not be as good a predictor of social phenotype in girls as in boys. In the overall sample, there was a significant relationship between social word use and ADOS-2 total scores (estimate: − 0.004, SE: 0.001, *z* = − 3.156, *p* = 0.002). However, there was no relationship between social word use and ADOS-2 RRB scores (estimate: − 0.001, SE: 0.002, *z* = − 0.46, *p* = 0.65), suggesting specificity within the social domain. For additional exploratory analyses predicting parent-rated phenotype, see Additional file 1: Supplemental Materials.

#### Exploratory analyses in NT

To determine whether increased social word production in girls was unique to autism, exploratory analyses were conducted in a smaller NT group (*N* = 34; 14 females) matched to the autistic group on age and IQ. After controlling for age (centered) and IQ (centered), a generalized linear regression model predicting participant social word production in the NT group revealed a significant main effect of sex (estimate: − 0.14, SE: 0.03, *z* = − 4.81, *p* < 0.001). Tukey-corrected pairwise comparisons of estimated marginal means revealed that the effect was driven by NT girls producing more social words than NT boys. Notably, this is the second study to show this pattern of results, wherein both autistic and NT girls use more social words than boys in semi-naturalistic conversations [34]. A GLM including age (centered), IQ (centered), and sex did not reveal a significant effect of sex on *friend* or *family* words. However, it is important to note that these null effects may be due to significantly reduced power in both the sample and the word subcategories (fewer words are included in *friend* and *family* word categories compared to the social word category). As such, these results should be considered preliminary and interpreted with caution.

## Discussion

In this study, we investigated sex differences in the behavioral presentation of autistic individuals by examining social word production during the interview sections of the ADOS-2 [77]. Our research question was motivated by prior work demonstrating that in NT adults, word choice and relative frequency can highlight what a speaker is attending to and finds important enough to describe [41, 42] and the theory that within autism, word choice can be a measure of social cognition or attention [44]. Our primary hypothesis was that autistic girls would use more social words than autistic boys (marking potentially increased social motivation, greater attentional focus on social groups, and/or camouflaging, but not necessarily greater social skill). This hypothesis was informed by previous research demonstrating sex differences in social motivation in autism (girls > boys) [28] and emerging research suggesting that autistic girls produce more socially-focused language than boys during narratives and unstructured interactions [30, 34]. Our results contribute to a growing literature that sharpens our conceptualization of autism in girls by characterizing subtle differences in conversational language.

A number of notable findings emerged: First, we found that autistic girls (*N* = 25) used significantly more social words than autistic boys during the interview section of the ADOS-2 Module 3 despite being matched on age, IQ, and autism symptom severity as rated by clinicians. Thus, despite the heterogeneity of autism, it is unlikely that the results of this study were driven by baseline sex differences in autism symptom severity. This result supported our primary hypothesis that autistic girls would use more social words compared to autistic boys and is broadly consistent with reports of sex-differentiated social phenotypes in autism. As others have noted, these differences may include increased social motivation and social focus (but not necessarily greater social skill) in autistic girls relative to autistic boys [26, 28, 34, 88].

Additionally, we found that social word use was related to clinical phenotype in the overall sample, such that heightened use of social words predicted fewer autism symptoms in the social affect domain of the ADOS-2. When examined separately in boys and girls, however, this result only held for boys. We interpret this finding with caution, as girls produced a consistently high number of social words (all girls produced more social words than 97.4% of boys, suggesting a possible ceiling effect), and the girls-only subsample was significantly smaller than the boys-only subsample. If this finding were to be replicated in a larger and more well-balanced sample, it could potentially indicate that the ADOS-2 Module 3 captures social communication differently for verbally fluent autistic school-aged boys than for girls. This is a critical consideration, as the norming sample used in the development of the ADOS-2 was predominantly male [78], leaving open the possibility that clinically meaningful sex differences in the behavioral presentation of autistic individuals went undetected or were judged unimportant for inclusion in the final algorithm. Understanding potential sex differences in the social language of school-age girls and boys on the ADOS-2 is a crucial step toward alleviating diagnostic disparities and facilitating opportunities for support and intervention, as many autistic individuals, particularly girls, are first diagnosed during this time [79, 80]. Accordingly, as suggested by others, diagnostic assessment should prioritize an in-depth understanding of an individual’s behavior across contexts and from multiple sources, rather than solely relying on a single cross-sectional assessment and score-thresholds on the “gold-standard” measures [105, 106].

It is important to note that the expert clinicians in our study detected social communication challenges in autistic girls *despite* elevated levels of social talk, suggesting recognition that using social words is not the same as demonstrating social skills or possessing social understanding. More concerning is the possibility that other adults who are not autism experts (e.g., teachers, primary care physicians, parents/caregivers) may observe increased social talk in autistic girls—compared to autistic boys—and interpret it as an indication of increased social competence, thus reducing the likelihood that girls are referred for an autism evaluation in the first place. Notably, although the autistic girls and boys in our sample were matched on clinician-rated autism symptoms, the girls had significantly higher SRS-2 scores across all subscales (of note, the SRS-2 is sex-normed). This pattern of results is consistent with prior research suggesting that the girls who ultimately meet criteria for autism on “gold-standard” diagnostic measures are more severely affected in real-world settings than autistic boys [107]. Thus, the autistic girls in our sample demonstrated both increased social talk and increased social challenges as rated by parents on the SRS-2 relative to NT girls.

Our second finding revealed important nuances in the *types* of social words produced by boys and girls, such that autistic girls were found to talk significantly more about *friends*. We did not directly measure friendship experiences in this study, but our results align with research demonstrating sex-differentiated friendship experiences in autism that are consistent with the friendship structures of NT girls and boys [27]. For example, given prior research, it is possible that autistic girls may talk more about *friends* because they are hyperaware of friends or social groups [34] and are more likely than boys to experience punishment or bullying from their peers when they misstep socially [27, 50]. This explanation fits with the results of qualitative research, wherein autistic girls report experiencing increased relational conflict from NT peers who punish them for “not getting it” socially by excluding them from the group or making them the butt of jokes [27, 58, 108]. In contrast, sex differences were not found for *family* category words; this is unsurprising given research suggesting generally typical levels of familial attachment in autistic children [109], and no evidence—to our knowledge—that autistic girls and boys are more or less focused on family during the school-aged years.

The overall pattern of results reported here could be interpreted in a variety of ways, all of which warrant future research. First, some researchers posit that autistic girls and women without ID may use intact cognitive processes to compensate for social difficulties by “masking” or “camouflaging” their autistic symptoms and actively working to appear non-autistic, leading them to present with better social skills than autistic boys and men [110–114]. Thus, autistic girls may be using more social words or talking about friends as a way to mirror their NT peers (who demonstrated a similar pattern of sex-differentiated social talk in the current study) or to improve their chances of fitting in. This interpretation is consistent with reports of greater effortful social compensation or masking by girls and women compared to boys and men on the spectrum that have been identified by prior research [21, 115]. Interestingly, mixed-methods research examining adolescents’ motivation for using camouflaging techniques has revealed that “making or keeping friends” was the most common theme reported [50]. From that perspective, autistic girls with heightened *friend* category word production might have learned to match NT levels of social talk about friends as a way to fit in with peers—thus partially “normalizing” the way they are perceived [30, 33, 34]. Exploring the *intentionality* with which social behaviors—including social words—are deployed by autistic girls could be accomplished using self-report questionnaires about camouflaging or masking, which have not yet been validated for children but have been used with adults [116].

Of note, autistic girls in our study did not have better social skills than autistic boys, as rated by clinicians, despite speaking differently. There are a number of possible explanations for this finding. First, autistic girls’ heightened social word use may reflect the influence of years-long exposure to gendered sociocultural norms, in which girls are expected to show more advanced interpersonal skills and focus more on social relationships compared to boys [117]. Research with NT girls has shown that they are socialized to participate in small, intimate groups with substantial language demands that value conforming to group interests, meaning that girls may be more likely to encounter peer situations that require more complex social skills [118]. Accordingly, elevated social word use in autistic and NT girls could be shaped by long-term exposure to societal messages and rewards—conveyed via the media, family, or peers—that steer girls toward social topics. In autism, elevated social word use may also act as a “social veneer” that makes girls sound more neurotypical while not necessarily indicating greater social skill. Future research designed to parse unique effects of culture and socialization on language in girls and boys is necessary to evaluate this potential explanation.

Notably, this is the second study to show that sex differences in social talk are not unique to autism; both autistic and NT girls produced significantly more social words than boys during the ADOS-2 and during a prior study of semi-naturalistic conversations [34]. This is a critical consideration, as heightened talk about social topics—and friends in particular—could complicate autism referral and diagnosis when observers expect a male-typical pattern of reduced social focus, which autistic girls do not always exhibit [119]. Rather, it may be more informative for potential referrers to consider to *how* girls are talking about social topics rather than *whether* or *how much* they are talking about social topics. Finally, elevated social word use could also reflect biological differences in social motivation that favor autistic girls [28], and which may contribute—in part—to a preponderance of boys diagnosed with the condition [18]. Importantly, high social motivation in autistic girls could be an area of strength leveraged by personalized social skills interventions. In all likelihood, the pattern of results reported in this study reflects a combination of the factors identified above. Future research should incorporate measures of masking or camouflaging, awareness of gender norms, gendered societal/familial influences, and social motivation to tease apart these complexities. For example, a study that examines whether camouflaging or social motivation—or a combination of the two—better predict social word use and whether sex moderates these associations, would help to clarify how social word use relates to autistic children’s cognition and social behavior. Ultimately, it is hoped that identifying differences in the female autistic profile will facilitate the development of services that are more responsive to the needs of girls and women on the spectrum [120].

### Limitations and future directions

This study has significant strengths, including a relatively large sample of well-matched, verbally fluent autistic girls and boys, but it also has several limitations. First, this sample was constrained to include verbally fluent children and adolescents aged 6–15 years, so our study results may not replicate in samples of younger children, adults, individuals who are not verbally fluent, or individuals with a VIQ below 70. Second, despite being one of the larger studies of conversational behavior in autism that utilizes direct behavioral assessment, the sample we report here is still small. Notably, due to the high rates of missed or misdiagnoses in autistic girls [18], it is unclear if the pattern of results we report will extend to the population of girls who are autistic but are not detected by currently available diagnostic methods and referral practices. Boys and girls in this sample were predominantly White and non-Hispanic, limiting our ability to assess how social language might differ in non-White and/or Hispanic children, and highlighting the need for future research in larger and more diverse cohorts. Additionally, given that language can be influenced by socioeconomic factors, future research on social language in autism should include measures that characterize SES and investigate potential relationships.

Clinicians in our study did not self-report race or level of enculturation, leaving an open question about the effect of same- versus difference-race dyads on social language use in autism. It is critical that future studies with very large samples be conducted with diverse participants and clinicians to examine potential ways in which the use of social words in autism may differ by race/ethnicity and enculturation match/mismatch. Children’s conversation partners in this study were primarily female clinicians, limiting our ability to assess patterns that might emerge during opposite-sex conversations in girls and same-sex conversations in boys [121]. Future studies with conversation partners of both sexes will explore how partners’ sex may affect the use of social words in autistic boys and girls.

Our methods and approach had several limitations as well. First, we examined the *number* of social, friendship, and family words produced during the ADOS-2. While valuable, such frequency-based analyses do not incorporate details about the contextual appropriateness of the words produced, which undoubtedly impact the effect they have during a conversation. Future iterations of the current research will explore social language in greater depth using qualitative approaches, enabling us to examine *nuances* in the social language of autistic girls and boys that could prove informative [81]. Second, we did not directly assess participants’ friendship experiences, so it is unclear whether or how individuals’ real-world peer relationships are related to the language they produced during the clinical assessment. Future studies should include measures of friendship insight and quality to examine potential interconnections between these variables. Third, we examined correlations between social word use and clinical phenotype as measured by ADOS-2 Total, SA, and RRB domain scores. These scores, while informative, were not designed to capture dimensional social phenotype and should be augmented by other measures such as behaviorally coded peer interactions, visual attention to social stimuli (eye tracking), or a targeted questionnaire about social interest and motivation. Future research should explore whether objective measures of social language, such as the one used in this study, map onto the third-party observational ratings of participants’ behavior used in most research. This area of investigation is particularly important, given the absence of significant effects of social word production on SRS-2, VABS, and SCQ scores in this study (see Supplemental Materials), and the field’s reliance on parent-report measures as a method for characterizing clinical phenotypes. Additionally, future research should also assess the impact of various therapeutic services (e.g., ABA, speech therapy, social skills training) on natural language measures.

Another limitation is that this study focused exclusively on the language produced by participants during ADOS-2 assessments. Although the ADOS-2 is a semi-structured, standardized assessment, it is possible that clinicians’ language may differ for boys and girls, either consciously or unconsciously. Future research should explore how clinicians’ language during the ADOS-2 could impact participants’ social language. Additionally, further research is needed to understand how clinicians’ preconceived notions about the social behavior of girls and boys may influence how they administer the ADOS-2. Understanding how the use of social words deployed during conversations differs in autistic children—and whether these differences are more prominent during formalized assessments or more relaxed conversational contexts with males/females—could shed light on the clinical heterogeneity currently complicating our efforts to effectively identify and diagnose children with autism, and ultimately to support their social development. Finally, the current study does not address the effect of *gender* on social language, as we utilized parent-reported assigned sex at birth to characterize participants. We recognize this approach does not account for individuals who identify as transgender, non-binary, or gender diverse and acknowledge the limitations of this methodology. Future research should examine language differences in a well-characterized and gender diverse sample, with the goal of understanding the complex intersecting effects of sex and gender on social behaviors like conversation.

## Conclusions

Natural language analytics hold great promise for generating high-dimensional, quantitative measures of clinically meaningful heterogeneity in the ~ 70% of individuals with autism who are verbally fluent. In this study, we examined one aspect of social behavior—language produced during the ADOS-2—and found that girls were significantly more likely than boys to use social words, and friend category words in particular. Sex differences in linguistic markers of social phenotype in autism are especially important in light of the late [8] or missed [18] diagnoses that disproportionately affect autistic girls. Specifically, heightened talk about social topics—including friends—could complicate autism referral and diagnosis when observers expect a male-typical pattern of reduced social focus, which autistic girls do not always exhibit [119]. When it comes to identifying autistic girls, our results suggest that the overall *amount* of talk about social topics is higher in autistic girls, and thus might not be a reliable marker for whether or not a girl should be referred for an expert assessment. Instead, *how* girls talk about social topics, and friends specifically, might be a better indicator of social functioning that could be used to guide referral decision-making and improve diagnostic accuracy [81]. Understanding and quantifying sex differences in natural language in autism will lead to more accurate phenotyping for boys *and* girls, which is necessary to improve early identification and inform personalized, sex-sensitive interventions that maximize long-term outcomes.

## Supplementary Information

**Additional file 1.** Additional exploratory analyses of participant social word production predicting parent-rated phenotype.

## Data Availability

The datasets generated and/or analyzed during the current study are not publicly available due to privacy concerns for minors with disabilities.

## Abbreviations

ADOS-2: Autism diagnostic observation schedule—2nd edition
ASC: Autism spectrum condition
EMM: Estimated marginal mean
IQ: Intelligence quotient
M: Mean
SCQ: Social Communication Questionnaire
VABS: Vineland Adaptive Behavior Scale
SD: Standard deviation
NT: Neurotypical
