# Supplementary material for: Friend matters: sex differences in social language during autism diagnostic interviews
Source: Mol Autism. 2022 Jan 10;13:5. doi: 10.1186/s13229-021-00483-1 (PMC8751321; doi:10.1186/s13229-021-00483-1)
Supplement: Supplementary file 1 — Additional file 1. Additional exploratory analyses of participant social word production predicting parent-rated phenotype. [file 13229_2021_483_MOESM1_ESM.docx]

**Supplemental Materials**

*Exploratory analyses predicting parent-rated phenotype.* To determine whether social word production was associated with parent-rated phenotype in autism, we modeled SRS-2, VABS, and SCQ scores as a function of social word production. First, we modeled SRS-2 Social Communication and Interaction (SCI) scores. After accounting for age (centered) and IQ (centered), social word production did not significantly predict SRS-2 SCI scores (est: .001, SE: .001, *z* = 1.38, *p* = .17). Next, we modeled SRS-2 Social Motivation scores as a function of social word production. After accounting for age (centered) and IQ (centered), social word production did not significantly predict SRS-2 Social Motivation scores (est: .001, SE: .001, *z* = 0.98, *p* = .32). We then explored the relationship between social word production and the VABS socialization domain score. After accounting for age (centered) and IQ (centered), there was not a significant effect of social word production on VABS socialization score (est: .001, SE: .001, *z* = .54, *p* = .59). Similarly, there was not a significant effect of social word production on SCQ scores (est: .001, SE: .001, *z* = .41, *p* = .68).

Importantly, there are notable differences in context of data collection for each of these measures compared to the ADOS-2 (which was the focus of our primary analyses). The ADOS-2 is a direct behavioral measure conducted by an expert clinician that occurs across a relatively brief time period in a controlled lab-based setting. In contrast, the SRS-2, VABS, and SCQ are parent-report measures of behaviors over the child’s lifespan across multiple settings. Additionally, in contrast to the ADOS-2, the SRS-2 and VABS are sex-normed measures. There were not significant differences between the VABS and SCQ scores of autistic girls and boys in our sample. However, although the autistic girls and boys in our sample were matched on clinician-rated autism symptoms measured by the ADOS-2, the girls had significantly higher SRS-2 scores across all subscales. This pattern of results is consistent with prior research demonstrating that girls who receive an autism diagnosis tend to show greater clinical impairment relative to same-sex NT peers as compared to boys, on various SRS-2 subscales [109].
